# Influence of S-PRG-based restorative and adhesive systems on biofilm formation and enamel demineralization in a simulated oral environment.

**DOI:** 10.1007/s00784-025-06731-5

**Published:** 2026-01-31

**Authors:** Luísa Figueredo de Carvalho, Maurício Malheiros Badaró, Sheila Cristina Stolf, Tamires Timm Maske, Glenda Ávila Marques, Laura Mello da Cunha, Júlia Silveira Longaray, Carla Lucía David Peña, Rafael Guerra Lund, Juliana Silva Ribeiro de Andrade

**Affiliations:** 1 Department of Dentistry, Federal University of Santa Catarina, Florianópolis, Santa Catarina Brazil; 2 Department of Preventive and Community Dentistry, Federal University of Rio Grande do Sul, Porto Alegre, Rio Grande do Sul Brazil; 3 Department of Restorative Dentistry, School of Dentistry, Federal University of Pelotas, Pelotas, Rio Grande do Sul Brazil; 4 Department of Cariology, Restorative Sciences, and Endodontics, School of Dentistry,, University of Michigan, 1011 North University Avenue, Ann Arbor, MI 48109 USA; 5 Department of Dentistry, Federal University of Santa Catarina, Av. Delfino Conti, Florianópolis, Santa Catarina 88040-900 Brazil

**Keywords:** Bioactive materials, S-PRG particles, Adhesive systems, Composite resin, Caries, Artificial oral cavity

## Abstract

**Objective:**

This study aimed to evaluate the impact of bioactive restorative materials on biofilm formation and cariogenic processes using an in vitro dynamic cariogenic biofilm model simulated by a Multifunctional Oral Cavity Simulator. The experimental design included composite resins and adhesive systems containing S-PRG particles (Beautifil and FL-Bond II – Shofu), compared against a conventional composite (Filtek Z350XT – 3 M ESPE) and a conventional adhesive system (Clearfil SE Bond – Kuraray).

**Methods:**

Samples were exposed to a controlled cariogenic environment and analyzed for colony-forming unit (CFU) counting, scanning electron microscopy (SEM), microhardness, and chemical modifications by Fourier-transform infrared spectroscopy (FTIR). One-way ANOVA and Tukey’s post hoc test was performed (α = 0.05).

**Results:**

CFU counting and SEM analysis revealed no significant differences in biofilm volume or microbial counts among groups (*P* > 0.05), indicating no reduction with bioactive materials. FTIR analysis showed a marked reduction in phosphate and carbonate absorption peaks compared to sound enamel, indicating a similar demineralization pattern regardless of material. Microhardness analysis revealed no significant differences among materials (*P* > 0.05).

**Conclusion:**

Our findings did not reveal superior protection of bioactive materials containing S-PRG particles against cariogenic challenges. In this context, more robust clinical evidence is still needed to confirm the effectiveness of bioactive materials in enhancing restoration longevity and caries control.

**Clinical significance:**

Under dynamic cariogenic conditions, S-PRG-based materials performed similarly to conventional restoratives materials. These findings indicate that the purported bioactivity may not result in measurable clinical benefits.

**Supplementary Information:**

The online version contains supplementary material available at 10.1007/s00784-025-06731-5.

## Introduction

The growing demand for minimally invasive dentistry has driven continuous advancements in resin composites and adhesive systems, aiming not only to restore dental function but also to exhibit bioactivity, such as promoting tissue regeneration, to enhance restoration longevity [1–3]. In this context, research on bioactive and regenerative dental materials has emerged, focusing on bioactive molecules and regenerative materials [1]. Particularly in restorative dentistry, biomaterials that promote biomineralization via bioactive ion release have become a focal point of research [4, 5]. Among the most prominent ions, fluoride plays a pivotal role by forming acid-resistant fluorapatite and inhibiting bacterial enzymes, thereby reducing caries and contributing to the longevity of restorations and improved oral health outcomes [6, 7].

Given this context, innovative strategies utilizing bioactive materials in direct restorative therapies have gained attention due to their potential to prevent caries-associated lesions. Giomer technology (SHOFU Inc., Kyoto, Japan) is a restorative concept based on Pre-Reacted Glass (PRG) particles, enabling the release of six ions—borate, aluminum, sodium, fluoride, strontium, and silicate [8]. This is achieved by incorporating pre-reacted fluoro-alumino-silicate glass filler with polyacrylic acid into the material matrix, enhancing its bioactive properties [7, 9, 10]. Utilizing the Surface Pre-Reacted Glass (S-PRG) mechanism, Giomers ensure sustained ion release and recharge, improving material performance and offering superior protection to tooth structure [11, 12]. Additionally, bioactive materials with S-PRG particles may outperform conventional adhesive systems and resin composites [13].

Although bioactive materials show promise in providing additional protective effects in dental restorations [7, 9, 12], there remains a gap in the literature regarding their advantages over conventional materials [5, 7, 9, 12]. While products such are marketed as offering the benefits of conventional materials, clinical evidence to validate these claims remains insufficient. In a recent systematic review and meta-analysis, Carvalho et al., 2025 [14] demonstrate no significant improvement in the longevity of posterior direct restorations when compared to conventional composites in randomized clinical trials.

Furthermore, these materials present inherent complexities and challenges when exposed to cariogenic environments, which are common in clinical settings. Dental adhesion is influenced by various biological and clinical factors [7, 15, 16]. Physiological changes, such as aging or responses to carious lesions, promote increased dentin mineralization [4]. This process reduces dentin permeability, negatively impacting the bond strength of adhesive systems [4]. Caries-affected dentin, located beneath decayed areas, presents unique characteristics; it is slightly demineralized, contains preserved collagen fibers, and has apatite crystals attached to these fibers, making it capable of remineralization [4, 17]. Therefore, the development of new adhesive systems that ensure a stable and long-lasting bond to both enamel and dentin, while promoting well-sealed margins and resistance to secondary caries, remains one of the greatest challenges in restorative dentistry [4, 17].

In this context, a deeper understanding of their mechanisms of action and potential effects on material properties and dental substrate responses is essential under these cariogenic conditions. Thus, this study aimed to evaluate the properties of these bioactive composites and adhesives using an in vitro cariogenic biofilm model based on a multifunctional oral cavity simulator (MOCS), which mimics clinical conditions for caries development [18]. The MOCS employed saliva-derived microcosm biofilms to replicate oral microbial complexity and dynamic nutrient flow, enhancing the clinical relevance of demineralization and biofilm formation [18]. By employing a dynamic microcosm biofilm within the MOCS, the present study seeks to address these methodological gaps and more closely simulate clinically meaningful cariogenic challenges. The null hypothesis tested was that bioactive materials do not offer protective benefits superior to conventional materials against the cariogenic challenge.

## Materials and methods

### Ethical aspects

The study protocol was reviewed and approved by the Research Ethics Committee on Human Subjects (CAAE: 43894821.7.0000.5347). Each volunteer received detailed information about the research procedures and provided written informed consent before participating.

### Sample size

The sample size was estimated based on previous studies [15, 19] to ensure statistically significant and reliable results in both biochemical and microbiological analyses. The following parameters were considered: a minimum detectable difference of 1.5 in the mean, a standard deviation of 0.5, a statistical power of 0.8, and a significance level of α = 0.05. These calculations were performed using statistical graphing software (SigmaPlot 14.0; Systat Software Inc., San Jose, CA, USA).

As a result, a sample size of n = 6 specimens per group was determined to be appropriate, totaling six experimental groups. This same sample size (*n* = 6) was used for all quantitative analyses, including colony-forming unit (CFU) counting, Fourier-transform infrared spectroscopy (FTIR), and microhardness evaluation, in accordance with methodologies previously applied in Maske et al. (2015) and Signori et al. (2021). Additionally, two specimens per group were allocated for qualitative SEM analysis. This clarification enhances transparency and reinforces reproducibility across all evaluated outcomes.

### In vitro experimental design

This study evaluated the properties of adhesives and composites containing S-PRG particles. To achieve this, the materials (composite resins and adhesive systems) were subjected to an in vitro cariogenic biofilm model based on a multifunctional oral cavity simulator. They were organized into experimental groups and analyzed for biofilm reduction through colony-forming unit (CFU) counts and scanning electron microscopy (SEM). The anticariogenic effects were assessed based on chemical substrate alterations, evaluated using Fourier-transform infrared (FTIR) spectroscopy and microhardness analysis. The materials used are detailed in Tables 1 and 2.

**Table 1:** – Resin materials used, including type, manufacturer, lot, and composition, according to the manufacturer’s instructions

Materials	Type	Manufacturer	Lot	Composition
Beautifil II LS	Giomer	Shofu Inc., Kyoto, Japan	032110	Bis-GMA, TEGDMA, Bis-MPEPP, S-PRG filler based on fluoroboroaluminosilicate glass, polymerization initiator, pigments, and other components.
Beautifil Bulk Restorative	Giomer	Shofu Inc., Kyoto, Japan	062263	Bis-GMA, TEGDMA, Bis-MPEPP, S-PRG filler based on fluoroboroaluminosilicate glass, polymerization initiator, pigments, and other components.
Filtek Z350XT Resin	Nanoparticulate Composite Resin	3 M ESPE, St. Paul, MN, USA	2,116,600,484	BIS-GMA, UDMA, TEGDMA, and BIS-EMA. Silica and zirconia particles.

*Bis-GMA Bisphenol A glycidyl methacrylate, *UDMA* Urethane dimethacrylate, *TEGDMA* Triethylene glycol dimethacrylate, *Bis-EMA* Ethoxylated bisphenol A dimethacrylate, *S-PRG* Pre-reacted glass ionomer, *Bis-MPEPP* Bisphenol A polyethylene glycol ether dimethacrylate

**Table 2:** Adhesive systems used, including type, manufacturer, lot, composition, and operating procedure according to the manufacturer’s instructions

Adhesive systems	Type	Manufacturer	Lot	Composition	Operating procedure
FL-Bond II	Two-step self-etching system	Shofu Inc., Kyoto, Japan	062201	Primer: Water, ethanol, carboxylic acid monomer, phosphoric acid monomer, and initiator. Adhesive: S-PRG-based adhesive, UDMA, TEGDMA, 2-HEMA, and initiator.	Apply the primer, wait for 10 s, and air dry. Apply the adhesive; do not dry. Light cure for 5s.
Clearfil SE Bond	Two-step self-etching system	Kuraray, Osaka, Japan	000200	Primer: MDP, HEMA, hydrophilic dimethacrylate, camphorquinone, and water. Adhesive: MDP, HEMA, Bis-GMA, hydrophobic dimethacrylate, N, N-diethanol p-toluidine, camphorquinone, silanized bonding agent, colloidal silica.	Air dry the dentin surface; Apply two coats of primer with gentle agitation for 20 s; Air dry for 20 s from a 20 cm distance; Apply an adhesive layer with gentle agitation for 20 s and light cure for 10s.

*MDP: 10-Methacryloyloxydecyl Dihydrogen Phosphate; HEMA: 2-Hydroxyethyl methacrylate; UDMA: Urethane dimethacrylate; S-PRG filler: Pre-reacted glass ionomer filler; TEGDMA: Triethylene glycol dimethacrylate; Bis-GMA: Bisphenol A glycidyl methacrylate.

For better organization of the analyses, the materials were divided into the following experimental groups (*n* = 6) (Table 3). The decision to use the control group with the Z350 XT composite resin (3 M ESPE, St. Paul, MN, USA) and the Clearfil SE Bond adhesive system (Kuraray, Osaka, Japan) was based on findings from the literature [20–22], which recognize these materials as gold-standard references due to their well-established clinical performance and high scientific rigor in previous studies.

**Table 3:** Experimental research groups

Groups	Abbreviation	Combination
Group 1	LsFl	Beautifil II LS Resin + FL-Bond II Adhesive
Group 2	LsCl	Beautifil II LS Resin + Clearfil SE Bond Adhesive
Group 3	BuFl	Beautifil Bulk Restorative Resin + FL-Bond II Adhesive
Group 4	BuCl	Beautifil Bulk Restorative Resin + Clearfil SE Bond Adhesive
Group 5	XTFl	Z350 XT Resin + FL-Bond II Adhesive
Group 6	XTCl	Z350 XT Resin + Clearfil SE Bond Adhesive

The experimental design did not include a positive control. Materials with potent antibacterial effects are not suitable for definitive restorations due to their mechanical limitations, solubility, and lack of long-term clinical indication [21, 23]. Their inclusion could artificially amplify the effect size and compromise the validity of the findings by creating a non-representative comparator [24]. Moreover, the biofilm model used in this study has been previously validated to discriminate antimicrobial performance among dental materials under reproducible conditions [25], ensuring the sensitivity of the system to detect potential differences between the experimental groups.

### Development of in vitro cariogenic biofilm

#### Preparation of teeth and samples

To evaluate the properties of the studied materials, adhesive restorations were fabricated as previously described by Signori et al. (2021) [19]. All research steps were performed by the same trained operator.

A total of 96 bovine teeth were selected based on visual inspection. Enamel-dentin discs were prepared with a diameter of 6 mm and a thickness of 2.5 mm, cut from the middle third using a diamond trephine bur at 400 rpm under irrigation. The obtained measurements were verified using a digital caliper. The disc surfaces were polished with sandpaper discs of different grit sizes (320, 600, and 1200). The discs were sectioned into two halves using a precision cutter (IsoMet™, Buehler Ltd., Lake Bluff, IL, USA). The bottom of each half was conditioned with 37% phosphoric acid for 15 s, followed by the application of an adhesive system according to the previously described groups, then light-cured following the manufacturer’s instructions using a VALO curing unit (Ultradent Products, South Jordan, UT, USA) at an intensity of 1000 mW/mm². Subsequently, each half was placed in a custom acrylic mold (6 mm in diameter and 3.5 mm in thickness) and restored with composite resin according to the pre-established groups. The resin was light-cured to obtain a cylindrical specimen with one half consisting of enamel-dentin and the other half featuring a composite resin surface, along with a bottom layer also made of composite resin.

**Fig. 1:**
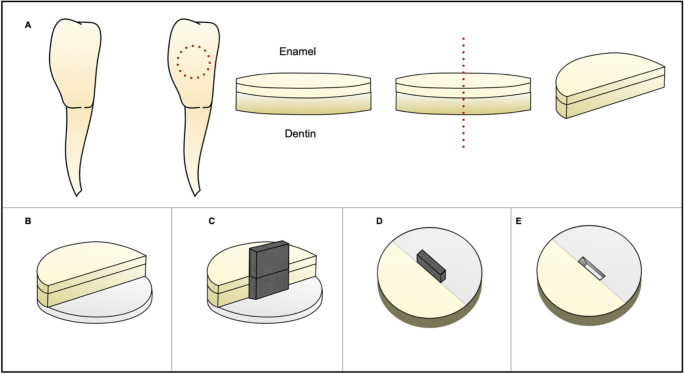
Schematic representation of the sample preparation with GAP. (**a**) Dentin-enamel discs were sectioned in half. (**b**) After acid etching and adhesive application on the lower dentin surface, one half was placed on a composite resin layer. (**c**) A spacer was positioned against the disc. (**d**) Composite resin was applied. (**e**) Final sample with interfacial gap formation

**Fig. 2:**
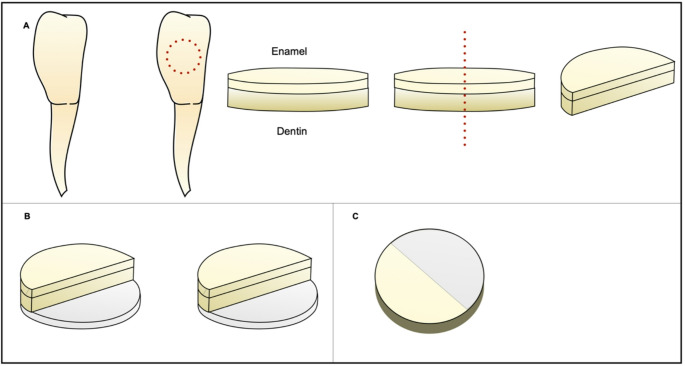
Schematic representation of the sample preparation without GAP. (**a**) Dentin-enamel discs were sectioned in half. (**b**) After acid etching and adhesive application on the lower dentin surface, one half was placed on a composite resin layer. (**c**) Composite resin was applied to the other half

To create retorations with interfacial gaps, a metallic spacer was inserted during composite resin placement. The spacer had a standardized width of 2.0 mm and a thickness of 0.1 mm and was positioned against the sectioned side of the disc to create an intentional gap between the composite resin and the tooth surface. Thus, the final gap dimensions were 2.0 mm in width, 2.5 mm in depth, and 0.1 mm in thickness. The spacer was not required for the FTIR spectroscopy analysis group, where the adhesive system was applied across the entire interface between the substrate and resin. After preparation, all samples were protected at the base and sides with nail varnish, leaving only the enamel surface exposed. This exposed enamel-resin region was polished to achieve a planar and highly glossy surface using abrasive polishing discs with different grits (Sof-Lex™ Pop-On, 3 M ESPE, St. Paul, MN, USA), with intermittent rinsing in distilled water to prevent powder accumulation on the surface and within the generated gap. Upon completion, the samples were sterilized with ethylene oxide and divided into six experimental groups. To enhance understanding of the sample preparation steps, a schematic diagram was created (Figs. 1 and 2).

### Multifunctional oral cavity simulator (MOCS)

#### Simulator description

To replicate a cariogenic challenge environment closer to reality, a Multifunctional Oral Cavity Simulator (MOCS) was employed. MOCS enables biofilm formation under dynamic conditions, closely mimicking key oral cavity features, including salivary flow, nutrient availability, and mechanical shear forces during biofilm development [18]. This system supports biofilm growth under fluctuating nutrient supply, simulating the natural cycles of nutrient scarcity, abundance, and metabolic byproduct clearance observed in the oral environment.

This arrangement consisted of three cylindrical chambers containing supports for sample attachment, positioned on a heating base set at 37 °C (± 2 °C). The chambers featured tubing that allowed the use of a specific culture medium and sucrose, facilitated by a peristaltic pump. The device simulated a controlled oxygen environment through a gas supply system for anaerobiosis (10% CO₂, 10% H₂, and 80% N₂), managed by a computer connected to peristaltic pumps for flow system control [18, 25].

#### Saliva collection

A total of 2.5 mL of fresh stimulated saliva was collected from a single volunteer using a paraffin film stimulation method for 10 min (Parafilm “M”, American National Can TM, Chicago, IL, USA). The saliva was obtained from a healthy volunteer (37 years old), who exhibited no signs of gingivitis or periodontitis and had not undergone antibiotic therapy for at least six months before collection. The volunteer refrained from oral hygiene for 24 h and from food intake for 2 h before the collection [18, 25].

#### Biofilm inoculation and growth

The previously collected saliva was homogenized using a vortex mixer (30 s), and added to 50 mL of McBain medium [18]. The solution was vortexed (30s) to obtain a final inoculum. The samples were positioned on holder-type supports within the simulator, and 3 mL of the inoculum (medium and saliva) was introduced through a specific inlet located above the sample holders, where it remained for 1 h to allow acquired pellicle formation. Following this period, the culture medium was perfused at an average flow rate of 0.06 mL/min. To establish a cariogenic challenge, the simulator performed intermittent 6-minute flows of 5% sucrose solution, applied three times daily at 2-hour intervals (average flow rate: 0.25 mL/min). This protocol enabled biofilm development under controlled anaerobic conditions (80% N₂, 10% CO₂, 10% H₂), maintained through a regulated gas flow of 0.06 N/mm^2^ for 1 min, applied twice daily, over 7 7-day experimental period [18]. The choice of 7 days follows the validated MOCS protocol, which reports that this period reliably generates quantifiable demineralization suitable for detecting material-related effects [18].

**Fig. 3:**
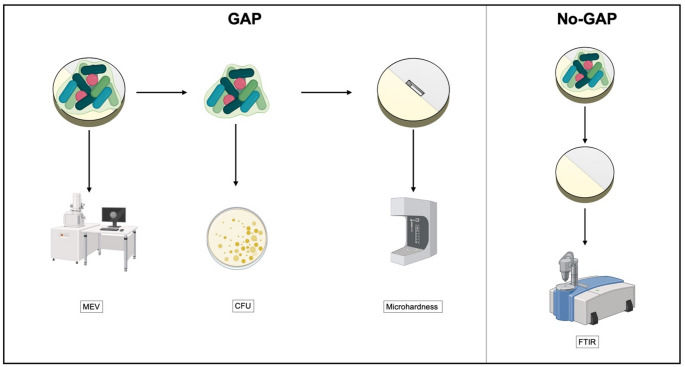
Experimental Flowchart

After the experimental time, the samples were removed for subsequent evaluations. Two samples from each experimental group were immediately fixed in 2.5% glutaraldehyde for later biofilm assessment using SEM. Biofilm from the remaining samples was collected to quantify biofilm reduction through CFU counts. Following biofilm removal, the samples underwent assessment of cariogenic effects via evaluation of chemical substrate alterations using FTIR spectroscopy and microhardness analysis. To facilitate understanding of the experimental procedures, a flowchart detailing the methodological steps was prepared (Fig. 3).

#### Scanning electron microscopy (SEM) analysis

To qualitatively analyze biofilm development on the samples, they were evaluated using SEM. For this purpose, after removal from the simulator, the samples were initially fixed in 2.5% glutaraldehyde for 24 h at 4 °C. Then, they underwent a gradual dehydration process with ethanol (10 min at 50%, 10 min at 60%, 10 min at 70%, 10 min at 80%, 10 min at 90%, and 10 min at 100%). Finally, hexamethyldisilazane (HMDS) was used, followed by gold-palladium coating. The images obtained from SEM (JEOL JSM 6390 LV, Akishima, Japan) were used to assess the volume of biofilm formation at magnifications up to 20,000x [26].

#### Biofilm collection and colony-forming counting

The biofilm formed on the surface was collected by standardized scraping using sterile plastic instruments and deposited into pre-weighed sterile microtubes (Eppendorf). The same procedure was applied to all specimens across all groups and was performed by a single trained operator to ensure methodological consistency. The wet weight of each collected biofilm was measured and then resuspended in 1mL of sterile saline. The suspension was sonicated using a probe (30s, 20 W) and serially diluted up to 10^7^ times (10^0^–10^7^) and inoculated in duplicate into the following culture media: Brain Heart Infusion Agar (BHI) for total microorganism quantification, Mitis Salivarius Agar (MSB) for visual evaluation of mutans streptococci, acidic BHI (pH 4.8) for acid-tolerant microorganisms, and Rogosa Agar for lactobacilli. The plates were incubated under anaerobic conditions at 37 °C for 96 h. After incubation, a trained operator performed CFU counting using an optical and stereoscopic microscope. The microbial counts were expressed as CFU mg⁻¹ of biofilm (wet weight) [19].

### Assessment of cariogenic effects

#### Enamel analysis by fourier transform infrared spectroscopy (FTIR)

After biofilm removal from the samples, they were analyzed for possible chemical alterations in the substrate following cariogenic challenge using ATR-FTIR spectroscopy (Shimadzu IR-Affinity-1, Japan), equipped with an attenuated total reflectance accessory. Spectra were obtained in absorbance mode using 32 co-added scans acquired over a frequency range of 600 to 4000 cm⁻¹ with 32 scans per sample. The phosphate absorbance peaks (between 900 and 1200 cm⁻¹) and carbonate groups (~ 872 cm⁻¹) were compared among experimental groups, with all spectra contrasted against sound enamel [15]. ATR-FTIR spectra were acquired by positioning the ATR crystal in direct contact with the enamel surface within the lesion area, in a standardized region adjacent to the restoration interface. This same region was analyzed for all specimens.

#### Microhardness analysis

**Fig. 4:**
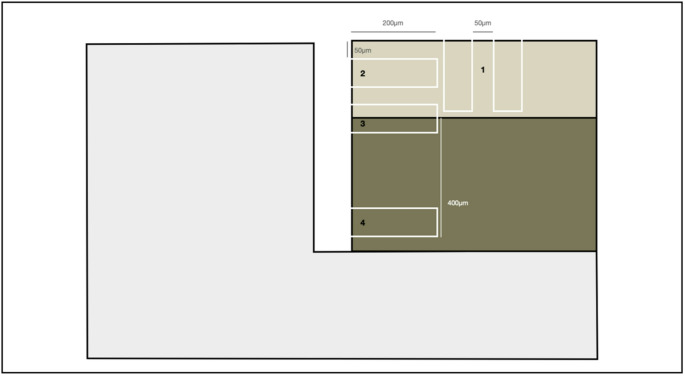
Diagram of the microhardness test

For microhardness analysis, the samples were sectioned using a precision cutter (IsoMet™, Lake Bluff, IL, USA), embedded in epoxy resin, and polished under wet conditions with silicon carbide abrasive papers of 600-, 1200-, 1500-, and 2000-grit, followed by final polishing with a 1 μm diamond suspension. Knoop microhardness measurements were performed on cross-sections using a microindenter (FM-700; FutureTech, Tokyo, Japan) to calculate the integrated demineralization area (ΔS) under a load of 25 g with a dwell time of 5 s for enamel and a load of 5 g for 5 s for dentin. Readings were taken at two points. The first set of readings was taken on the enamel surface at a distance of 200 μm from the GAP, with 2 rows of indentations spaced 50 μm apart (enamel 1 and enamel 2. The second set of readings was performed on the opposite wall of the GAP: at 50 μm from the surface for enamel readings, at the enamel-dentin junction (EDJ), and at 400 μm from the EDJ for dentin readings (Fig. 4). All measurements were conducted by a blinded, trained operator [15, 19]). ΔS data were calculated by subtracting the hardness profile (Knoop hardness number, kgf mm − 2) of the demineralized area from the hardness values obtained from sound enamel or dentin.

#### Statistical analyses

Statistical analysis was performed using GraphPad Prism version 10 (GraphPad Software, San Diego, CA, USA). For the ΔS analysis, data from “enamel 1” and “enamel 2” measurements were averaged to obtain a single enamel value per specimen for statistical analysis. Initially, data were assessed for variance homogeneity using Levene’s test and for normality using the Shapiro-Wilk test to ensure assumptions were met for subsequent analyses. Comparisons between groups were conducted using one-way analysis of variance (One-way ANOVA), followed by Tukey’s test for multiple comparisons. A significance level of 5% was adopted.

## Results

### Biofilm formation analysis via SEM

**Fig. 5:**
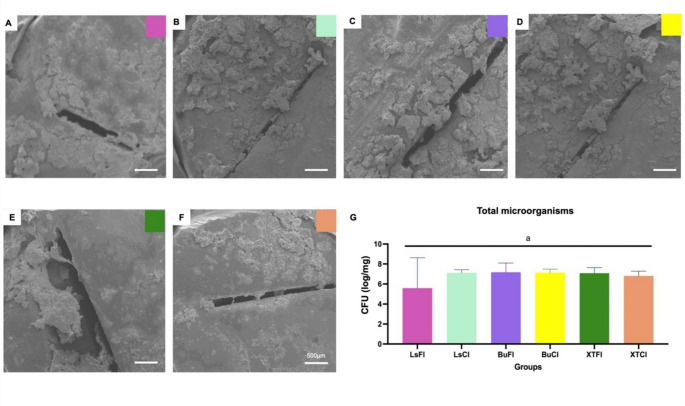
**(A-F)** SEM images of biofilm formation on the groups: (**A**) Beautifil II LS Resin + FL-Bond II Adhesive (LsFl); (**B**) Beautifil II LS Resin + Clearfil SE Bond Adhesive (LsCl); (**C**) Beautifil Bulk Restorative Resin + FL-Bond II Adhesive (BuFl); (**D**) Beautifil Bulk Restorative Resin + Clearfil SE Bond Adhesive (BuCl); (**E**) Z350 XT Resin + FL-Bond II Adhesive (XTFl); (**F**) Z350 XT Resin + Clearfil SE Bond Adhesive (XTCl); (**G**) Representation of total microorganism formation results among the groups (*p* > 0.05)

The SEM images (Fig. 5) revealed significant biofilm formation on all experimental groups. No substantial reduction in biofilm volume was observed in groups where bioactive materials were used. The composition of the tested materials did not significantly impact microbial adhesion and proliferation under the adopted experimental conditions.

### Colony-forming unit counting

**Fig. 6:**
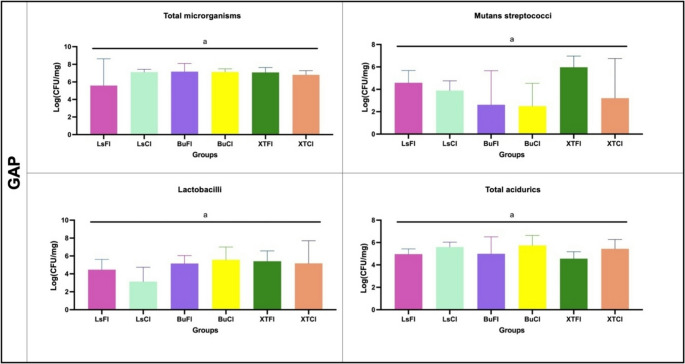
Colony-forming unit (CFU) counts for each group presenting a GAP, evaluated for total microorganisms, mutans streptococci, total acidurics and lactobacilli. No statistically significant differences were observed among the groups (*p* > 0.05)

**Fig. 7:**
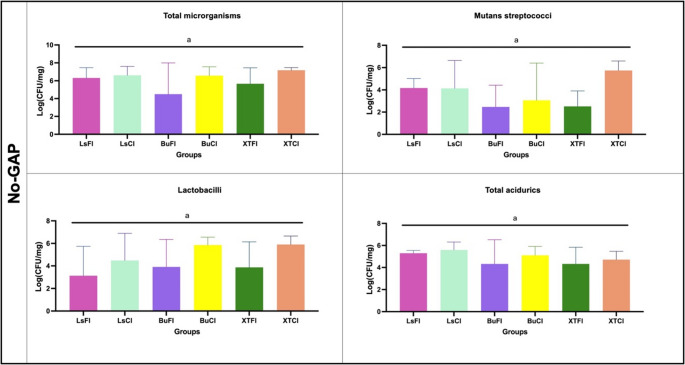
Colony-forming unit (CFU) counts for each group without GAP, evaluated for total microorganisms, mutans streptococci, total acidurics and lactobacilli. No statistically significant differences were observed among the groups (*p* > 0.05)

Microbiological analysis results showed that for all evaluated microorganisms, including total microorganisms, mutans streptococci, *aciduric bacteria*, and lactobacilli., no statistically significant differences were observed among the analyzed groups (*P* > 0.05), regardless of the presence or absence of the GAP in the samples. These findings are detailed in Figs. 6 and 7. CFU log results (mean ± standard deviation) for all microbial outcomes are presented in Appendix Table 1.

### Chemical analysis of the substrate after cariogenic challenge - fourier transform infrared spectroscopy (FTIR)

**Fig. 8:**
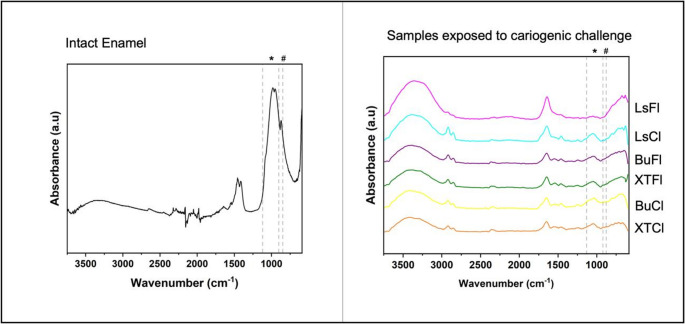
FTIR spectra of sound dental enamel and samples after exposure to the cariogenic challenge.*Note:* Asterisks (*) denote characteristic calcium and hashtag (#) indicate phosphate peaks

FTIR spectra (Fig. 8) revealed characteristic absorbance peaks associated with phosphate (PO₄³⁻) and carbonate (CO₃²⁻) groups in different enamel samples, including those subjected to the cariogenic challenge (LsFI, LsCI, BuFI, XTFI, BuCI, XTCI) and sound enamel. Spectroscopy indicated that the absorbance peaks corresponding to phosphate (between 900 and 1200 cm⁻¹) and carbonate (~ 872 cm⁻¹) did not show differences among the analyzed groups. However, a significant difference in peak intensity was observed when compared to sound enamel, indicating changes in enamel mineral composition. As the FTIR assessment was qualitative and not normalized or quantified, these findings indicate mineral degradation but do not allow numerical comparison of demineralization among materials.

### Analysis of enamel and dentin microhardness alterations after cariogenic challenge

**Fig. 9:**
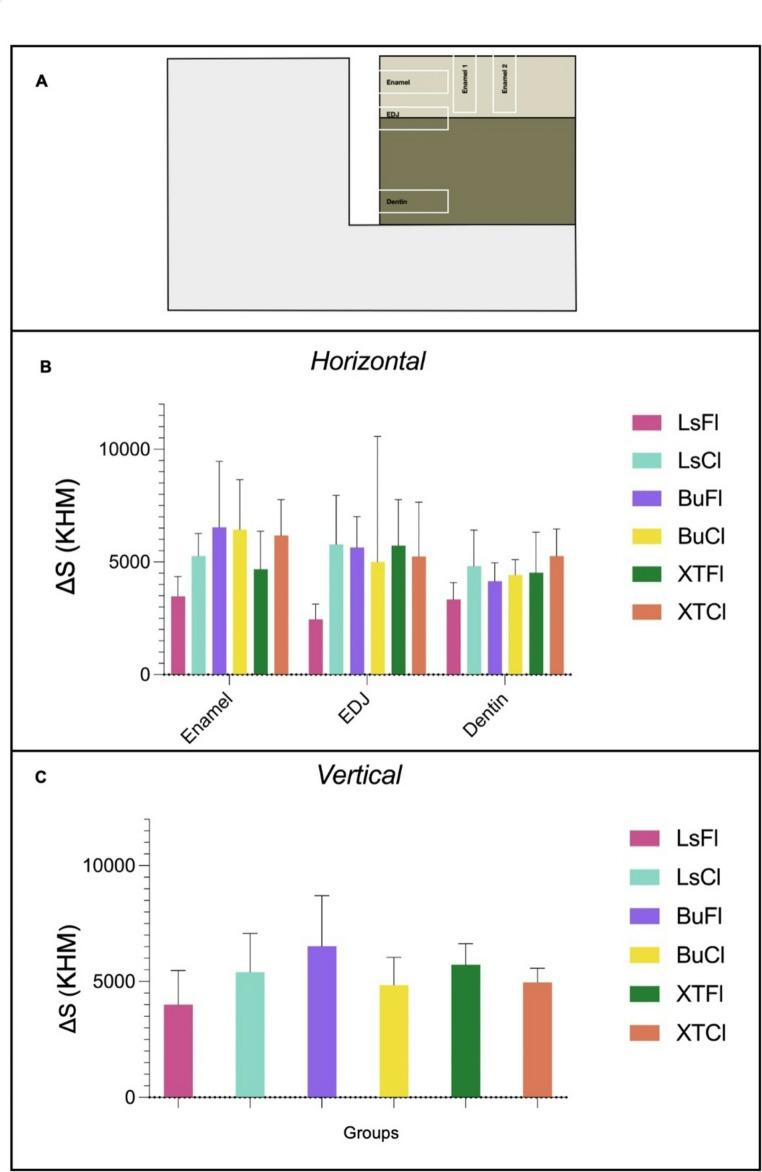
Microhardness analysis for groups presenting a GAP. Values are reported as ΔS (integrated microhardness loss) and expressed as mean ± standard deviation. (**A**) Diagram of the microhardness test; (**B**) Horizontal ΔS across the GAP, including enamel, dentin, and the enamel-dentin junction (EDJ). No statistically significant differences were observed among the groups (*p* > 0.05); (**C**) Vertical ΔS values for enamel (Enamel 1 and Enamel 2 location were averaged). No statistically significant differences were observed among the groups (*p* > 0.05)

Regarding microhardness, the analysis results for each research group are presented in Fig. 9. Similarly to the microbiological analysis, no statistically significant differences were observed among the groups under any evaluated conditions (*P* > 0.05). In the horizontal analysis, average microhardness values were obtained for enamel, dentin, and the enamel-dentin junction (EDJ), with no significant variations between the analyzed regions. In the vertical analysis, the two enamel measurement sites (“Enamel 1” and “Enamel 2”) corresponded to repeated points within the same standardized region and were therefore averaged to obtain a single enamel value per specimen for statistical analysis, as they did not represent distinct distances from the restoration interface. Similarly, no significant differences were observed among experimental groups in the vertical assessment.

## Discussion

Contemporary restorative dentistry continues to prioritize the development of minimally invasive, durable materials that combine structural restoration with biological functionality. This pursuit is driven by the need to mitigate complications such as secondary caries, a leading cause of restorative failure linked to cariogenic biofilm accumulation at marginal gaps. These gaps, often resulting from polymerization shrinkage of composites or degradation of the adhesive layer, facilitate bacterial microinfiltration and acidic byproduct diffusion, accelerating demineralization and interface breakdown [27–29]. Preserving the biological and chemical integrity of the hybrid layer at the tooth-restoration interface is thus critical to preventing lesions and enhancing restorative longevity [29–31].

To thoroughly address these issues, the present study sought to replicate the complex oral cavity environment using an artificial mouth simulator under a complex microcosm model and controlled gaps to evaluate biofilm formation and the progression of carious lesions [25, 27, 30, 31]. The biological challenges involved include the simulation of multispecies biofilms, which better represent the microbial diversity found in vivo [19, 29]. Although bioactivity represents a promising advancement in restorative dentistry, its clinical potential still depends on overcoming challenges related to efficient ion release and the maintenance of mechanical properties. The results underscore the complexity of developing effective bioactive restoratives, as no significant differences in protective benefits against cariogenic challenges were observed between bioactive and conventional materials. Consequently, the null hypothesis, positing no superiority of bioactive materials in this context, is confirmed.

According to Wuersching et al. (2025) [29], marginal gaps larger than 60 μm facilitate the development of secondary caries, especially under masticatory loads that promote fluid exchange and bacterial colonization. Furthermore, the choice of bacterial model is crucial, as mixed biofilms composed of multiple species more accurately represent oral microbial dynamics than single-species models, such as *Mutans streptococci* [18, 25]. The metabolic activity of the biofilm is influenced by the oral environment, which regulates bacterial changes [19, 27]. The cariogenic challenge favors the growth and maintenance of bacteria capable of surviving in an acidic environment [25, 29].

Microbiological analyses revealed no statistically significant differences in biofilm reduction among the evaluated groups, irrespective of the use of bioactive materials (in various forms) or conventional restorative materials. This was consistent across all tested microorganisms, including total microorganisms, *mutans streptococci*, *aciduric bacteria*, and *total aciduric spp*., with no significant variations observed between groups (*P* > 0.05), even when considering the presence or absence of GAP in the samples (Figs. 6 and 7). These findings were further corroborated by qualitative scanning electron microscopy (SEM) images, which showed no visual distinctions in biofilm formation. Importantly, the results align with recent systematic reviews, such as that by de Carvalho et al.. (2025) [14], which highlights the lack of significant advantages in restoration longevity when comparing bioactive restorative materials to conventional resin composites.

Furthermore, FTIR analysis revealed a significant reduction in the intensity of phosphate (~ 1030–1100 cm⁻¹) and carbonate (~ 870 cm⁻¹) characteristic peaks, corroborating the findings of Maske et al. (2015) and indicating enamel demineralization after the cariogenic challenge. This decrease in spectral peaks reflects mineral content loss, indicating the dissolution of hydroxyapatite, the primary constituent of enamel [32]. When proposing remineralization, it is expected that the inorganic portion lost during demineralization, particularly carbonate, be replenished, as observed in the intact enamel spectrum (Fig. 8), where the carbonate band at ~ 870 cm⁻¹ is evident. However, none of the tested materials demonstrated restoration of this inorganic signal. Moreover, when comparing the inorganic fraction relative to the CH band (~ 2800 cm⁻¹), no increase in the inorganic component was observed. On the contrary, the proximity of these bands suggests that remineralization was not promoted by any of the bioactive materials tested, in agreement with microhardness findings [33]. It is important to note that FTIR data were derived from a qualitative, non-normalized assessment, and therefore support the presence of mineral degradation but do not allow quantitative comparison of demineralization magnitude among groups.

The microhardness analysis of enamel and dentin revealed no statistically significant differences (*P* > 0.05) between the experimental groups under any evaluated conditions. Horizontal evaluations (Fig. 9), which assessed enamel, dentin, and the enamel-dentin junction (EDJ), demonstrated comparable average microhardness values across these regions, with no notable variations between groups. Likewise, vertical evaluations (Fig. 9), focusing on two distinct areas of the enamel surface, also showed no significant differences in microhardness among the groups. Importantly, microhardness outcomes in this study represent the post-challenge condition of the tissues and were interpreted based on ΔS, which compares the demineralized profile with the sound enamel or dentin values obtained from the same specimen. Therefore, the resulting values do not reflect the intrinsic hardness differences between enamel and dentin, but rather the magnitude of mineral loss induced by the cariogenic environment. These results align with the microbiological findings, further corroborating the equivalence between bioactive and conventional materials. Collectively, the data indicate that the bioactive materials did not confer additional protection against the applied cariogenic challenge, as evidenced by the absence of significant microhardness preservation in either horizontal or vertical assessments.

This result suggests a limitation in remineralization in the face of the cariogenic challenge [32, 34]. Enamel ionic regulation is a dynamic process dependent on ion diffusion in the oral environment; however, the significant reduction indicates that the remineralizing capacity of the evaluated materials was insufficient to prevent enamel degradation [33, 34].

Based on these results, it is hypothesized that S-PRG particles become immobilized due to the low permeability of the highly cross-linked polymer networks, which prevents the diffusion of ions [35, 36]. Notably, these particles appeared entrapped in the dense polymer network, which likely restricted their bioactive ion release. This immobilization may explain the observed lack of bioactivity, specifically, the absence of biofilm reduction, failure to inhibit enamel demineralization, and inability to promote enamel remineralization following cariogenic challenge. Clinically, the reduced tooth-restoration interface further restricts fluid contact necessary for ion dispersion, potentially rendering released ion concentrations insufficient to exert bioactive effects [37, 38]. Thus, optimizing ion release without hampering mechanical performance remains a critical challenge for bioactive restorative materials.

This study offers valuable contributions to understanding bioactive materials under a cariogenic challenge in a complex environment. However, it is fundamental to acknowledge the inherent complexities and limitations of laboratory (in vitro) studies compared to clinical trials, particularly concerning the simulation of dynamic conditions and ionic recharge present in the oral cavity. Our results establish a proof of concept for the initial anti-biofilm action, as well as in vitro microhardness and chemical changes. Furthermore, although the interfacial gap was standardized using a 0.1 mm metallic spacer, the post-polishing gap dimension was not directly verified, which should be considered a methodological limitation. In addition, although the agar-plate method was used to microorganism counts, no taxonomic profiling of the microcosm community was performed using molecular methods, limiting conclusions regarding microbial composition and ecological shifts. Although the sample size was previously determined and focused on demineralization values, a post hoc power assessment indicated that the design was sufficiently powered to detect moderate-to-large differences in CFU outcomes; therefore, very subtle microbiological differences between materials cannot be entirely ruled out, and the microbiological results should be interpreted with caution.

To advance, future studies should focus on determining the stability and durability of bioactive activity, associating it with critical mechanical properties such as fracture resistance and wear behavior over longer periods. Additionally, optimizing ion release profiles and evaluating the possibility of recharging these bioactive ions will be crucial to meet biological and structural demands [18, 30]. Ultimately, translating bioactive potential into clinical efficacy will require a multidisciplinary approach that harmonizes innovative materials science with rigorous biological validation.

## Conclusions

Based on the findings of this study, bioactive restorative materials containing S-PRG particles did not demonstrate superior performance in preventing demineralization or modulating cariogenic biofilm formation when compared to conventional materials, under simulated oral conditions. These results suggest that the claimed bioactivity of such materials may not translate into measurable clinical benefits in short-term scenarios or without specific activation conditions. Clinicians should therefore exercise caution when relying solely on the marketed bioactive potential of restorative materials. Further long-term studies, including in vivo models and varying environmental challenges, are necessary to better understand the real impact of bioactive formulations in restorative dentistry.

## Supplementary Information

Below is the link to the electronic supplementary material.

Supplementary Material 1 (DOCX 17.6 KB) 

Supplementary Material 2 (DOCX 16.7 KB)

## Data Availability

No datasets were generated or analysed during the current study.

## References

[1] Iftikhar S, Jahanzeb N, Saleem M, Ur Rehman S, Matinlinna JP, Khan AS (2021) The trends of dental biomaterials research and future directions: a mapping review. Saudi Dent J 33:229–238. 10.1016/j.sdentj.2021.01.002 34194185 10.1016/j.sdentj.2021.01.002 PMC8236547

[2] Almulhim KS, Syed MR, Alqahtani N, Alamoudi M, Khan M, Ahmed SZ, Khan AS (2022) Bioactive inorganic materials for dental applications: a narrative review. Materials 15:6864. 10.3390/ma15196864 36234205 10.3390/ma15196864 PMC9573037

[3] Costa MP, Giacomini MC, Zabeu GS, Mosquim V, Dallavilla GG, Santos PSDS, Wang L (2024) Impact of functional monomers, bioactive particles, and HEMA on the adhesive performance of self-etch adhesive systems applied to simulated altered dentin. J Dent 151:105379. 10.1016/j.jdent.2024.105379 39341447 10.1016/j.jdent.2024.105379

[4] Perdigão J (2020) Current perspectives on dental adhesion: (1) dentin adhesion – not there yet. Jpn Dent Sci Rev. 10.1016/j.jdsr.2020.08.004 34188727 10.1016/j.jdsr.2020.08.004 PMC8216299

[5] Melo MAS, Mokeem L, Sun J (2022) Bioactive restorative dental materials—the new frontier. Dent Clin North Am 66:551–566. 10.1016/j.cden.2022.05.005 36216446 10.1016/j.cden.2022.05.005

[6] Ikemura K, Tay FR, Endo T, Pashley DH (2008) A review of chemical-approach and ultramorphological studies on the development of fluoride-releasing dental adhesives comprising new pre-reacted glass ionomer (PRG) fillers. Dent Mater J 27:315–339. 10.4012/dmj.27.315 18717159 10.4012/dmj.27.315

[7] Gonulol N, Ozer S, Tunc ES (2014) Water sorption, solubility, and color stability of Giomer restoratives. J Esthet Restor Dent 27:300–306. 10.1111/jerd.12119 25145876 10.1111/jerd.12119

[8] Pimentel ES, França FMG, Turssi CP, Basting RT, Vieira-Junior WF (2023) Effects of in vitro erosion on surface texture, microhardness, and color stability of resin composite with S-PRG fillers. Clin Oral Investig 27:3545–3556. 10.1007/s00784-023-04968-6 36995429 10.1007/s00784-023-04968-6

[9] Francois P, Fouquet V, Attal JP, Dursun E (2020) Commercially available fluoride-releasing restorative materials: a review and a proposal for classification. Materials 13:2313. 10.3390/ma13102313 32443424 10.3390/ma13102313 PMC7287768

[10] Sajini S, Mushayt A, Almutairi T, Abuljadayel R (2022) Color stability of bioactive restorative materials after immersion in various media. J Int Soc Prev Community Dent 12:418. 10.4103/jispcd.JISPCD_40_22 36312581 10.4103/jispcd.JISPCD_40_22 PMC9615936

[11] Ozer F, Patel R, Yip J, Yakymiv O, Saleh N, Blatz MB (2022) Five-year clinical performance of two fluoride-releasing Giomer resin materials in occlusal restorations. J Esthet Restor Dent 34:1213–1220. 10.1111/jerd.12948 35934807 10.1111/jerd.12948

[12] Shimizu S, Kotake H, Takagaki T, Shinno K, Miyata S, Burrow MF, Hotta M, Nikaido T (2021) Evaluation of bonding performance and multi-ion release of S-PRG filler containing self-adhesive resin composite. Dent Mater J 40:1257–1263. 10.4012/dmj.2020-142 34193722 10.4012/dmj.2020-142

[13] Zan KW, Nakamura K, Hamba H, Sadr A, Nikaido T, Tagami J (2018) Micro-computed tomography assessment of root dentin around fluoride-releasing restorations after demineralization/remineralization. Eur J Oral Sci 126:390–399. 10.1111/eos.12558 30055024 10.1111/eos.12558

[14] Carvalho LF, Gimenes E Silva M, Barboza ADS, Badaró MM, Stolf SC, Cuevas-Suárez CE, Lund RG, de Ribeiro Andra JS (2025) Effectiveness of bioactive resin materials in preventing secondary caries and retention loss in direct posterior restorations: a systematic review and meta-analysis. J Dent 152:105460. 10.1016/j.jdent.2024.105460 39547467 10.1016/j.jdent.2024.105460

[15] Maske TT, solan CP, van de Sande FH, Peixoto AC, Faria-E-Silva AL, Cenci MS, Moraes RR (2015) A biofilm cariogenic challenge model for dentin demineralization and dentin bonding analysis. Clin Oral Investig 19:1047–1053. 10.1007/s00784-014-1331-1 25323985 10.1007/s00784-014-1331-1

[16] Garoushi S, Vallittu PK, Lassila L (2018) Characterization of fluoride releasing restorative dental materials. Dent Mater J 37:293–300. 10.4012/dmj.2017-161 29279547 10.4012/dmj.2017-161

[17] Tavares FVB, Maciel CM, Watanabe MU, Vieira-Junior WF, França FMG, Turssi CP, Basting RT, Influence of adhesive systems containing different functional monomers on the longevity of bond strength at different dentin depths. Int J Adhes Adhes. 132:103723., Maske TT, Cenci MS, Patzlaff R et al (2024) 2025. Presentation of a new multifunctional oral cavity simulator: the MOCS. Braz Oral Res. 39:e022. 10.1590/1807-3107bor-2025.vol39.022 10.1590/1807-3107bor-2025.vol39.022 PMC11844815 40008731

[18] Maske TT, Cenci MS, Patzlaff R, et al., 2025. Presentation of a new multifunctional oral cavity simulator: the "MOCS". Braz Oral Res. 39:e022. 10.1590/1807-3107bor-2025.vol39.022 10.1590/1807-3107bor-2025.vol39.022 PMC11844815 40008731

[19] Signori C, Maske TT, Digmayer Romero VH, Cenci MS (2021) Influence of biofilm removal from the tooth-restoration interface on the progression of secondary caries lesions: A preliminary in vitro model study. Biofouling 1–12. 10.1080/08927014.2020.1870219 10.1080/08927014.2020.1870219 33401970

[20] Fehrenbach J, Isolan CP, Münchow EA (2021) Is the presence of 10-MDP associated with higher bonding performance for self-etching adhesive systems? A meta-analysis of in vitro studies. Dent Mater 37:1463–1485. 10.1016/j.dental.2021.08.014 34456050 10.1016/j.dental.2021.08.014

[21] Panetta A, Alshami R, Watson E, Patel N (2024) Evaluating glass ionomer cement longevity in the primary and permanent teeth—an umbrella review. J. Funct. Biomater 15(2):48. 10.3390/jfb15020048 38391901 10.3390/jfb15020048 PMC10890125

[22] Mézquita-Rodrigo I, Scougall-Vilchis RJ, Moyaho-Bernal MA, Rodríguez-Vilchis LE, Rubio-Rosas E, Contreras-Bulnes R (2021) Using self-etch adhesive agents with pit and fissure sealants. Eur Arch Paediatr Dent 23:233–241. 10.1007/s40368-021-00655-w 34365570 10.1007/s40368-021-00655-w PMC8349235

[23] Burke FJT, Lucarotti PSK (2018) The ultimate guide to restoration longevity in England and Wales. Part 3: glass ionomer restorations – time to next intervention and to extraction of the restored tooth. Br Dent J 224:865–874. 10.1038/sj.bdj.2018.436 29855590 10.1038/sj.bdj.2018.436

[24] Melo MAS, Garcia IM, Alluhaidan T, Qaw M, Montoya C, Orrego S, Balhaddad AA, Mokeem L (2025) The next frontier in antibacterial dental resins: a 20-year journey of innovation and expectations. Dent Mater 41(9):1045–1057. 10.1016/j.dental.2025.06.013 40527702 10.1016/j.dental.2025.06.013

[25] Maske TT, van de Sande FH, Arthur RA, Huysmans MCDNJM, Cenci MS (2017) In vitro biofilm models to study dental caries: a systematic review. Biofouling 33:661–675. 10.1080/08927014.2017.1354248 28792234 10.1080/08927014.2017.1354248

[26] Takamizawa T, Imai A, Hirokane E, Tsujimoto A, Barkmeier WW, Erickson RL, Latta MA, Miyazaki M (2019) SEM observation of novel characteristics of the dentin bond interfaces of universal adhesives. Dent Mater 35:1791–1804. 10.1016/j.dental.2019.10.006 31727447 10.1016/j.dental.2019.10.006

[27] Ferracane JL (2017) Models of caries formation around dental composite restorations. J Dent Res 96:364–371. 10.1177/0022034516683395 28318391 10.1177/0022034516683395 PMC5384487

[28] Demarco FF, Collares AC, Coelho-de-Souza FC et al (2022) Longevity of composite restorations is definitely not only about materials. Dent Mater 39:1–12. 10.1016/j.dental.2022.11.009 36494241 10.1016/j.dental.2022.11.009

[29] Wuersching SN, Kohl L, Hickel R, Schwendicke F, Kollmuss M (2025) Assessing the marginal seal of bioactive restorative materials in class II cavities with a bacterial penetration model. Dent Mater 41:553–560. 10.1016/j.dental.2025.03.003 40074569 10.1016/j.dental.2025.03.003

[30] Gauthier R, Aboulleil H, Chenal JM, Chevalier J, Colon P, Grosgogeat B (2021) Consideration of dental tissues and composite mechanical properties in secondary caries development: a critical review. J Adhes Dent 23:297–308. 10.3290/j.jad.b1649941 34269540 10.3290/j.jad.b1649941

[31] Askar H, Krois J, Göstemeyer G, Bottenberg P, Zero D, Banerjee A, Schwendicke F (2020) Secondary caries: what is it, and how it can be controlled, detected, and managed? Clin Oral Investig 24:1869–1876. 10.1007/s00784-020-03268-7 32300980 10.1007/s00784-020-03268-7

[32] Lubarsky GV, D’Sa RA, Deb S, Meenan BJ, Lemoine P (2012) The role of enamel proteins in protecting mature human enamel against acidic environments: a double layer force spectroscopy study. Biointerphases 7:14. 10.1007/s13758-011-0014-6 22589057 10.1007/s13758-011-0014-6 PMC4875143

[33] Orilisi G, Tosco V, Monterubbianesi R, Notarstefano V, Özcan M, Putignano A, Orsini G (2021) ATR-FTIR, EDS and SEM evaluations of enamel structure after treatment with hydrogen peroxide bleaching agents loaded with nano-hydroxyapatite particles. PeerJ e10606. 10.7717/peerj.10606 33575125 10.7717/peerj.10606 PMC7849511

[34] Maske TT, Isolan CP, van de Sande FH, Peixoto AC, Faria-E-Silva AL, Cenci MS, Moraes RR (2012) A biofilm cariogenic challenge model for dentin demineralization and dentin bonding analysis. Clin Oral Investig 19:1047–1053. 10.1007/s00784-014-1331-1 10.1007/s00784-014-1331-1 25323985

[35] Par M, Spanovic N, Bjelovucic R, Marovic D, Schmalz G, Gamulin O, Tarle Z (2019) Long-term water sorption and solubility of experimental bioactive composites based on amorphous calcium phosphate and bioactive glass. Dent Mater J 38:555–564. 10.4012/dmj.2018-145 30713282 10.4012/dmj.2018-145

[36] Trinca RB, Vela BF, Dos Santos Vilela H, Braga RR (2024) Ion release mechanisms in composites containing cap particles and hydrophilic monomers. Dent Mater 40:1047–1055. 10.1016/j.dental.2024.05.008 38772841 10.1016/j.dental.2024.05.008

[37] Schmalz G, Hickel R, Price RB, Platt JA (2023) Bioactivity of dental restorative materials: FDI policy statement. Int Dent J 73:21–27. 10.1016/j.identj.2022.11.012 36577639 10.1016/j.identj.2022.11.012 PMC9875272

[38] Campos AL, Vela BF, Pires Silva Borges L, Trinca RB, Pfeifer CS, Braga RR (2023) Compositional boundaries for functional dental composites containing calcium orthophosphate particles. J Mech Behav Biomed Mater 144:105928. 10.1016/j.jmbbm.2023.105928 37302206 10.1016/j.jmbbm.2023.105928 PMC10330647

