# Supplementary material for: Influence of S-PRG-based restorative and adhesive systems on biofilm formation and enamel demineralization in a simulated oral environment
Source: Clin Oral Investig. 2026 Jan 31;30(2):67. doi: 10.1007/s00784-025-06731-5 (PMC12858577; doi:10.1007/s00784-025-06731-5)
Supplement: Supplementary file 1 — Supplementary Material 1 (DOCX 17.6 KB) [file 784_2025_6731_MOESM1_ESM.docx]

**Appendix Table A**

**CFU (log/mg biofilm) data from GAP samples (Mean ± SD)**

| **Group** | **Total microorganisms**  **(Mean ± SD)** | **Mutans streptococci (Mean ± SD)** | **Total acidurics (Mean ± SD)** | **Lactobacilli. (Mean ± SD)** |
| --- | --- | --- | --- | --- |
| LsFl | 5.16 ± 3.2 | 4.59 ± 1.22 | 4.99 ± 0.52 | 4.41 ± 1.28 |
| LsCl | 7.11 ± 0.31 | 3.89 ± 0.88 | 5.6 ± 0.44 | 3.13 ± 1.61 |
| BuFl | 7.17 ± 0.93 | 2.62 ± 3.05 | 5.0 ± 1.51 | 5.16 ± 0.89 |
| BuCl | 7.13 ± 0.36 | 2.5 ± 2.04 | 5.74 ± 0.9 | 5.56 ± 1.45 |
| XTFl | 7.08 ± 0.56 | 5.97 ± 1.0 | 4.56 ± 0.62 | 5.41 ± 1.15 |
| XTCl | 6.8 ± 0.47 | 3.21 ± 3.53 | 5.44 ± 0.84 | 5.17 ± 2.54 |

| **Group Group** | **Total microorganisms**  **(Mean ± SD)** | **Mutans streptococci (Mean ± SD)** | **Total acidurics (Mean ± SD)** | **Lactobacilli*.* (Mean ± SD)** |
| --- | --- | --- | --- | --- |
| LsFl | 6.31 ± 1.16 | 4.17 ± 0.85 | 5.3 ± 0.25 | 3.13 ± 2.61 |
| LsCl | 6.61 ± 1.0 | 4.14 ± 2.5 | 5.6 ± 0.71 | 4.48 ± 2.41 |
| BuFl | 4.5 ± 3.5 | 2.47 ± 1.96 | 4.32 ± 2.2 | 3.91 ± 2.45 |
| BuCl | 6.57 ± 0.99 | 3.05 ± 3.35 | 5.11 ± 0.8 | 5.85 ± 0.7 |
| XTFl | 5.66 ± 1.78 | 2.51 ± 1.4 | 4.33 ± 1.51 | 3.87 ± 2.27 |
| XTCl | 7.18 ± 0.28 | 5.74 ± 0.86 | 4.71 ± 0.76 | 5.91 ± 0.75 |

**CFU (log/mg biofilm) data from No-GAP(Mean ± SD)**
