# Supplementary material for: Influence of S-PRG-based restorative and adhesive systems on biofilm formation and enamel demineralization in a simulated oral environment
Source: Clin Oral Investig. 2026 Jan 31;30(2):67. doi: 10.1007/s00784-025-06731-5 (PMC12858577; doi:10.1007/s00784-025-06731-5)
Supplement: Supplementary file 2 — Supplementary Material 2 (DOCX 16.7 KB) [file 784_2025_6731_MOESM2_ESM.docx]

**Appendix B**

Mean ΔS (Integrated Demineralization Area) ± SD for horizontal microhardness analysis across groups.

| **Group** | **Enamel (Mean ± SD)** | **EDJ (Mean ± SD)** | **Dentin (Mean ± SD)** |
| --- | --- | --- | --- |
| LsFl | 3933 ± 1374.81 | 2237 ± 813.81 | 3169.5 ± 782.80 |
| LsCl | 5084.5 ± 996.65 | 5787.67 ± 1935.98 | 4629.83 ± 1501.44 |
| BuFl | 7133.72 ± 2997.26 | 5458.33 ± 1307.76 | 4272.33 ± 801.76 |
| BuCl | 6351.17 ± 1992.59 | 4597.67 ± 5082.02 | 4195.67 ± 826.53 |
| XTFl | 4694.33 ± 1516.39 | 5624.33 ± 1840.49 | 4658.83 ± 1646.78 |
| XTCl | 6452.67 ± 1571.75 | 5250.17 ± 2153.68 | 5108.83 ± 1138.81 |

Mean ΔS (Integrated Demineralization Area) ± SD for vertical microhardness analysis across groups.

| **Group** | **Enamel 1 (Mean ± SD)** | **Enamel 2 (Mean ± SD)** |
| --- | --- | --- |
| LsFl | 3795 ± 1170.66 | 4205.83 ± 2816.49 |
| LsCl | 5603.83 ± 2892.80 | 5197.33 ± 998.95 |
| BuFl | 7705.5 ± 2863.72 | 5349.33 ± 3626.46 |
| BuCl | 4933.5 ± 859.26 | 4764.5 ± 1553.65 |
| XTFl | 6060 ± 1649.05 | 5390.83 ± 902.33 |
| XTCl | 4747.67 ± 946.84 | 5187.83 ± 827.05 |
